# Efficacy of Electroacupuncture Therapy in Patients With Postherpetic Neuralgia: Study Protocol for a Multicentre, Randomized, Controlled, Assessor-Blinded Trial

**DOI:** 10.3389/fmed.2021.624797

**Published:** 2021-05-21

**Authors:** Hantong Hu, Yejing Shen, Xinwei Li, Hongfang Tian, XingLing Li, Yang Li, Yingying Cheng, Lei Wu, Dexiong Han

**Affiliations:** ^1^Department of Acupuncture and Moxibustion, The Third Affiliated Hospital of Zhejiang Chinese Medical University, Hangzhou, China; ^2^Department of Acupuncture and Moxibustion, Lishui Hospital of Traditional Chinese Medicine, Lishui, China; ^3^Department of Acupuncture and Moxibustion, Tongde Hospital of Zhejiang Province, Hangzhou, China

**Keywords:** electroacupuncture, postherpetic neuralgia, pain, randomized controlled trial, protocol

## Abstract

**Introduction:** The efficacy of conventional treatments for treating postherpetic neuralgia (PHN) remains unsatisfactory. Thus, this multicentre, randomized controlled, assessor-blinded trial aims to investigate the efficacy and safety of electroacupuncture (EA) therapy in patients with PHN.

**Methods and Analysis:** This multicentre randomized controlled trial will enroll 132 patients with PHN from 3 hospitals. All patients will be randomly assigned to either the EA combined with medication group or medication group through a computerized central randomization system in a 1:1 ratio. Outcome measures will be assessed before intervention, at 2, 4, 6 weeks after intervention and at the end of 8-week follow-up. Primary outcomes will be sensory thresholds and pain intensity. Secondary outcomes will include dosage of analgetic, quality of life, anxiety, and depression severity and sleep quality. All adverse effects will be assessed during the trial.

**Conclusions:** This study will provide evidence to ascertain whether EA is effective and safe for treating PHN.

**Ethics and Dissemination:** Ethics approval (No.ZSLL-KY-2017-025) has been obtained from the Ethics Committee of The Third Affiliated Hospital of Zhejiang Chinese Medical University. Informed consent will be signed prior to subject enrolment. The results will be submitted to international peer-reviewed journals and presented at international conferences.

**Trial Registration Number:** The study protocol has been registered in the clinicaltrials registry with the identification code NCT04594226.

## Introduction

Postherpetic neuralgia (PHN) is the most common sequela of herpes zoster (HZ) (1). It is generally defined as pain in the skin lesion area lasting for more than 1 month after the healing of HZ rash (2). PHN is characterized by persistent or intermittent pain (3), which can be spontaneous or induced. And it is often accompanied by local paresthesia, anxiety, sleep disorders, and other symptoms, which seriously affect the quality of life in patients.

Although multiple kinds of therapies have been adopted to treat PHN, the efficacy of conventional therapies (e.g., medication, local anesthesia, surgical operations) remains unsatisfactory because many of these therapies can cause side effects leading to patient intolerance (4). In addition, many of these therapies are not applicable to all patients with PHN and some patients have high-risk for recurrence. Therefore, PHN has been a worldwide health challenge and many clinicians and patients are seeking for help from complementary and alternative medicine.

As one of the most widely used complementary therapies for treating a large spectrum of diseases for thousands of years, acupuncture has advantages of simple operation and less side effects, thereby is gaining increasing popularity in the world (5). A great number of clinical trials have revealed the efficacy of acupuncture on a variety of pain conditions (6, 7), including PHN (8), but the reliability of most trials is not so robust against methodological defects, such as small sample size, absence of randomization (9).

To date, there is no high quality evidence regarding the therapeutic effect of acupuncture for treating PHN (10). Thus, this multicentre randomized controlled trial (RCT) is designed to investigate the efficacy and safety of electroacupuncture (EA) for treating PHN.

## Method and Analysis

### Study Design

**Figure 1:**
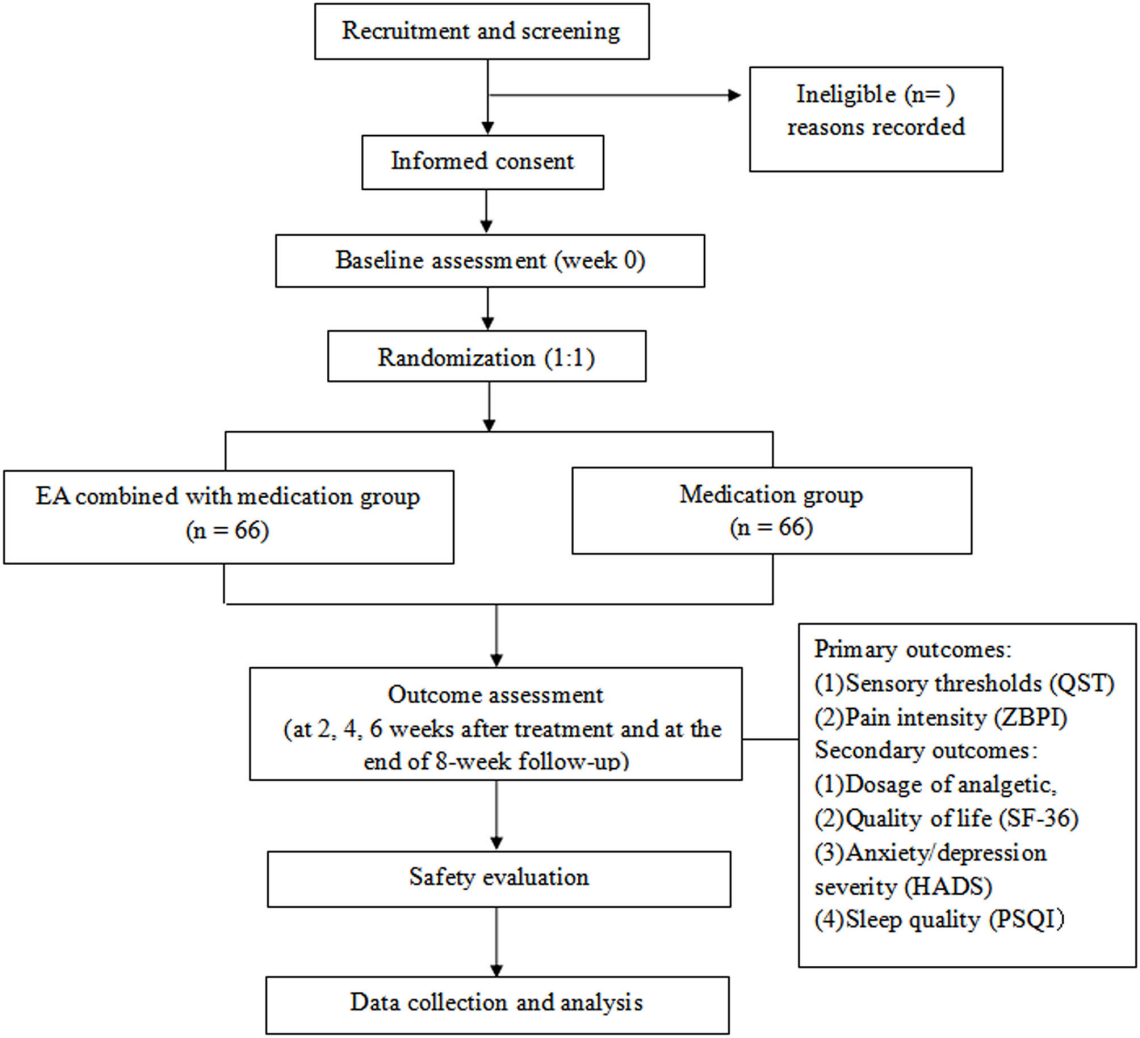
Flow chart of the study process. EA, electroacupuncture; HADS, Hospital Anxiety and Depression Scale; PSQI, Pittsburgh Sleep Quality Index; QST, Quantitative Sensory Testing; SF-36, Short Form 36 Health Survey; ZBPI, Zoster Brief Pain Inventory.

This multicentre trial will adopt a randomized controlled and assessor-blinded design. Eligible participants will be randomly assigned to the EA combined with medication group or medication group in a 1:1 allocation ratio. Flow chart of the study process is shown in Figure 1 and the trial schedule of enrolment, treatments and assessments is displayed in Table 1. Participants will receive 6 weeks of treatments in two groups: EA combined with medication, medication. The reporting of this protocol is based on the Standards for Reporting Interventions in Clinical Trials of Acupuncture (STRICTA) (11) and the SPIRIT reporting guidelines (12).

**Table 1:** Schedule of enrolment, treatments, and assessments.

**Study period**	**Screening**	**Baseline**	**Treatment period**	**Treatment period**	**Treatment period**	**Follow-up period**
		**week 0**	**Week 2**	**Week 4**	**Week 6**	**Week 14**
Eligibility screening	○					
Demographic data	○					
Case data	○					
Inclusion criteria	○					
Exclusion criteria	○					
Informed consent	○					
Treatment			○	○	○	
**Outcome assessment**						
(1) QST		○	○	○	○	○
(2) ZBPI		○	○	○	○	○
(3) Dosage of analgetic		○	○	○	○	○
(4) SF-36		○	○	○	○	○
(5) HADS		○	○	○	○	○
(6) PSQI		○	○	○	○	○
Safety assessment			○	○	○	○
Recurrence of PHN						○

*○, required; HADS, Hospital Anxiety and Depression Scale; PHN, postherpetic neuralgia; PSQI, Pittsburgh Sleep Quality Index; QST, Quantitative Sensory Testing; SF-36, Short Form 36 Health Survey; ZBPI, Zoster Brief Pain Inventory*.

### Participant Enrollment

Participants will be enrolled from 3 sub-centers, including the Third Affiliated Hospital of Zhejiang Chinese Medical University, Tongde Hospital of Zhejiang Province, and Lishui Hospital of Traditional Chinese Medicine. Enrollment methods will mainly include advertisements in local newspapers and health-related TV programmes, and posters in communities and hospitals. The eligibility of participants will be evaluated by the researchers based on the criteria described in details as follows.

### Inclusion and Exclusion Criteria

The inclusion and exclusion criteria are summarized in details in Table 2.

**Table 2:** The inclusion criteria and exclusion criteria of PHN patients.

**Inclusion criteria**	**Exclusion criteria**
(1) Patients have a medical history of HZ; (2) Skin lesion has healed in the region of HZ, but the duration of posterior neuralgia persists for more than 30 days; (3) 20 ≤ age ≤ 80 years, male or female; (4) Participants can fully understand the study protocol and a written informed consent is signed.	(1) Acute HZ or HZ has not disappeared; (2) HZ belongs to a special type, such as ophthalmic HZ, auricular HZ, HZ that involves internal organs, meningeal HZ, and disseminated HZ; (3) Pregnant or lactating women; (4) Patients have severe complications in cardiovascular, cerebrovascular, liver, kidney, hematopoietic and other systems, or have malignant tumor, mental illness, immune deficiency, hemorrhagic disorders and other diseases; (5) Patients have severe cognitive impairment and cannot understand the study protocol; (6) Patients cannot receive EA treatment due to any reasons. (7) Patients are currently taking antithrombotic drugs.

*EA, Electro-acupuncture; HZ, Herpes Zoster; PHN, Postherpetic Neuralgia*.

### Discontinuation Criteria

(1) Subjects have serious adverse reactions during the study and it is not appropriate to continue participating in the trial;
(2) Subjects have serious complications or deteriorating conditions during the study and emergency measures are urgently needed;
(3) Subjects ask to quit the trial halfway;
(4) Subjects have poor compliance and cannot comply with the study protocol.

### Elimination Criteria

(1) Subjects don't meet the inclusion criteria but they are included in the trial by mistake;
(2) Subjects don't follow the prescribed treatment or have incomplete data that affects the efficacy evaluation and safety evaluation;
(3) Subjects with poor compliance and withdraw from the study by themselves;
(4) Subjects received adjunctive treatments other than the intervention of this trial.

### Randomization and Allocation Concealment

All patients will be randomly assigned to either the EA combined with medication group or medication group via a computerized central randomization system. If a participant is enrolled, an independent researcher in each center, who has no contact with participants, acupuncturists and outcome assessors, will log into the computerized central randomization system to generate random sequence, dividing eligible participants into two groups in a ratio of 1:1. The researcher will make random allocation cards, each with its group allocation information and sealed into an opaque envelope, which will not be open until the first treatment.

### Blinding

Given to the characteristics of acupuncture, acupuncture manipulators will not be blinded. Nevertheless, the treatment, outcome assessment, and statistical analysis will be performed by different researchers independently. Outcome assessment will be performed by assessors blinded to the allocation information. Statistical analyses will be performed by statisticians blinded to the allocation information.

### Intervention

Participants will receive either EA combined with medication or medication alone.

The treatment course of both groups is up to 6 weeks and the follow-up period is 8 weeks.

#### EA Combined With Medication Group

Patients in this group received EA combined with gabapentin. Gabapentin is administrated as described in 2.8.2. Acupuncture will be performed with needles in the specification of 0.30 × 40 mm (Suzhou Medical Products Factory Co., Ltd, China). EA procedures will be performed with electronic needle instruments (Hans-100A, Nanjing Jisheng Medical Technology Co., Ltd, China).

##### (1) Acupoint Selection

The location standard of acupoints is based on the National Standard Nomenclature and Location of Acupuncture Points 2006 (GB/T12346-2006). Locations of acupoints applied in this trial are summarized in Table 3.

**Table 3:** Location and indication of acupoints for treating PHN.

**Acupoints**	**Location**	**Indication**
**Primary acupoints**		
(1) Ashi acupoints	Ashi points are defined as the most painful points in the herpes zoster lesion are.	**–**
(2) Jiaji acupints (EX-B2)	Jiaji acupoints on the ipsilateral side will be selected according to the corresponding ganglia involved by herpes zoster. Jiaji acupoints (EX-B2) are located 0.5 cun bilateral to the posterior midline in the dorsal thoracic and lumbar region, ranging from the first thoracic vertebra to the fifth lumbar vertebra.	–
(3) Xi-cleft points		
Waiqiu (GB36)	On the lateral aspect of the lower leg, 7 cun above the tip of the external malleolus, on the anterior border of the fibula, at the level of GB 35.	For pain sites that mainly distributed in the gallbladder meridian
Liangqiu (ST34)	When the knee is flexed, on the anterior aspect of the thigh, on the line connecting the anterior superior iliac spine and the lower lateral border of the patella, 2 cun above the patella.	For pain sites that mainly distributed in the stomach meridian
Jinmen (BL63)	On the lateral aspect of the foot, directly below the anterior border of the external malleolus, lateral to the lower border of the cuboid bone.	For pain sites that mainly distributed in the bladder meridian
Wenliu (LI7)	With the elbow flexed, the point is on the dorsal radial side of the forearm, 5 cun above the wrist crease	For pain sites that mainly distributed in the large intestine meridian
**Supplementary acupoints**		
Xingjian (LR2)	On the dorsum of the foot, proximal to the margin of the web between the 1st and 2nd toes, at the junction of the red and white skin.	For stagnated heat of the liver meridian
Yinlingquan (SP9)	On the medial aspect of the lower leg, in the depression of the lower border of the medial condyle of the tibia.	For damp heat of the spleen meridian
Geshu (BL17)	On the back, 1.5 cun lateral to the lower border of the spinous process of the 7th thoracic vertebra.	For blood stasis obstruction in vessels
Sanyinjiao (SP6)	On the medial aspect of the lower leg, 3 cun above the medial malleolus, on the posterior border of the medial aspect of the tibia.	For Yin deficiency of liver and kidney

Primary acupoints include Ashi points, Jiaji acupoints (EX-B2) and Xi-cleft acupoints. Ashi points are defined as the most painful points in the HZ lesion area and the selection number of Ashi points will be controlled within 3 for each treatment session. Jiaji acupoints (EX-B2) on the ipsilateral side will be selected according to the corresponding ganglia involved by HZ. Xi-cleft acupoints will be selected based on the distribution regions of PHN. For pain sites that mainly distributed in the gallbladder meridian, Waiqiu (GB36) will be selected. For pain sites that mainly distributed in the stomach meridian, Liangqiu (ST34) will be selected. For pain sites that mainly distributed in the bladder meridian, Jinmen (BL63) will be selected. For pain sites that mainly distributed in the large intestine meridian, Wenliu (LI7) will be selected.

Supplementary acupoints will be chosen based on TCM syndrome differentiation. For stagnated heat of the liver meridian, Xingjian (LR2) will be selected. For damp heat of the spleen meridian, Yinlingquan (SP9) will be selected. For blood stasis obstruction in vessels, Geshu (BL17) will be selected. For Yin deficiency of liver and kidney, Sanyinjiao (SP6) will be selected.

##### (2) Operation

Patients are asked to select the appropriate body position in terms of HZ sites, such as lateral, supine and prone positions, etc. The skin around the selected acupoint is routinely disinfected. Patients are then given acupuncture and deqi sensation is achieved in all acupoints. Two paired of acupoints (i.e., two Jiaji acupoints and two Ashi points, respectively) are connected to the EA apparatus. EA parameter is selected as dilatational wave and frequency is 2/100 Hz. EA intensity is determined according to patients' endurance.

Needles will be retained and EA will last for 30 min for each session. Patients will receive a total number of 30 EA sessions, with the frequency of 5 sessions per week for 6 weeks.

#### Medication Group

Participants in the medication group will only receive oral administration of gabapentin capsule. The dosage of gabapentin starts from 100 mg Tid. 100–300 mg can be increased every 3–7 days and taken in three doses, but the total amount is not more than 1,800 mg/day. The dosage of gabapentin will be adjusted according to the patient's condition until his pain is effectively controlled.

After maintaining the medication for 2 weeks, the dosage can be gradually reduced and 100 mg can be reduced every 3–7 days.

### Outcome Measures

#### Primary Outcome Measures

(1) Change in sensory perception measured by quantitative sensory testing (QST);
  QST is a psychophysical test method that investigates the functional state of the somatosensory system by means of calibrated stimuli and subjective perception thresholds (13). It is a valid and reliable test method that is often adopted in trials involving HZ or PHN (14, 15). QST will be performed in accordance with the protocol developed by the German ResearchNetwork on Neuropathic Pain (DFNS) that was described in details elsewhere (16) and in brief, multiple tests will be performed as supplied in Appendix 1.
(2) Change in pain intensity measured by Zoster Brief Pain Inventory (ZBPI) instrument
  ZBPI is a herpes zoster specific questionnaire to quantify pain and discomfort in HZ and PHN patients. The validity and reliability of the ZBPI as an outcome measure for HZ or PHN has been proved (17), so it is frequently used in trials involving PHN patients (18, 19). The detailed questionnaire of ZBPI is supplied in Appendix 2.

Primary outcomes will be assessed before intervention, at 2, 4, 6 weeks after intervention and at the end of 8-week follow-up.

#### Secondary Outcome Measures

(1) Change in dosage of prescription analgetic;
(2) Change in quality of life measure by 36-item short form health survey (SF-36);
(3) Change in anxiety and depression severity measure by hospital anxiety and depression scale (HADS);
(4) Change in sleep quality measured by Pittsburgh sleep quality index (PSQI)

Secondary outcomes will be assessed before intervention, at 2, 4, 6 weeks after intervention and at the end of 8-week follow-up.

### Safety Evaluation

The indicator of safety evaluation is the frequency of adverse events (AEs) arisen during the treatment course. For this trial, AEs related to acupuncture and EA mainly include unbearable pain, bleeding, haematoma, skin pigmentation, skin allergies, fainting/sweating/dizziness during acupuncture, or other uncomfortable symptoms. AEs related to medication mainly include gastrointestinal discomfort and drug allergies. AEs that occur throughout the study will be assessed and recorded and by the investigators. Investigators should also record the occurrence date, degree, duration, and treatment measures of AEs.

At occurrence of AEs, emergency measures will be taken in a timely manner. For serious AEs, the researchers should report to the principal investigator and ethics committee immediately, who will determine whether the participant should be withdrawn from the study.

### Quality Control

Prior to the beginning of enrollment, in order to avoid treatment bias induced by different understanding of acupuncture methods from different researchers, one experienced acupuncturist will be selected from each sub-center, who should have at least 5 years of acupuncture clinical experience, to receive intensive training on acupuncture treatment procedure based on the treatment regimen. Meanwhile, all outcome assessors will attend standard training to practice outcome assessments and fill in the case report forms uniformly.

Throughout the trial, all study data will be collected in the case report forms and double entered into the electronic data capture system by independent researchers in due time. Dropouts or withdrawals from the trial will be recorded in details. A data safety monitoring committee according to the guidance of Data Safety Monitoring Board will be employed. Staffs in the committee will be responsible for monitoring the data and have the right to reveal blinded data. They will also verify the authenticity between the raw data and the recorded data. Besides, the principal investigator and other research team members from different centers will meet every 3 month to discuss issues arisen during the trial the best solutions.

Lastly, in order to promote enrollment and participant compliance, all treatment cost and all outcome measurements will be free for participants.

### Sample Size Estimation

The participants will be assigned to 2 groups in a ratio of 1:1. The sample size is calculated based on the change of ZBPI from baseline to 6 weeks after intervention, the results of which are acquired from our pilot study. It is assumed that n_1_ = n_2_, α = 0.05, and β = 0.2. Thus, each group approximately requires 57 participants. With an estimated dropout rate of 15%, a total of 132 subjects are needed, with 66 participants in each group.

### Statistical Analysis

Statistical analysis will be performed using SPSS 17.0 for Windows (SPSS Inc., Chicago, IL, USA) by third-party statisticians. For categorical data, it will be displayed as counts and percentages. Numeric data with normal distribution will be expressed as mean ± standard deviations, whereas data with skewed distribution as median with 95% confidence intervals. Between-group differences for dichotomous variables for the baseline characteristic, such as gender and ages, will be compared according to the Chi-square test. For data in normal distribution, repeated measures analysis of variance (ANOVA) will be used to perform within-group and between-group comparison by assessing change in continuous variables before and after intervention at different time-points. For data in skewed distribution, within-group and between-group comparison will be assessed using non-parametric test. A *P* value of <0.05 will be considered statistically significant.

### Ethical Approval and Study Registration

Ethics approval (approval document No.ZSLL-KY-2017-025) has been obtained from the Ethics Committee of The Third Affiliated Hospital of Zhejiang Chinese Medical University. Prior to enrollment, researchers will explain the objective, research items, benefits and potential risks of the study to participants in details. All participants will be entitled to full rights to decide whether they are willing to participate in this trial. An informed consent form should be signed before inclusion in this trial. Throughout out the study, participant privacy will be protected strictly. All personal and disease information of participants will be kept confidential.

The study protocol has been registered in the clinicaltrials registry with the identification code NCT04594226.

## Discussion

### The Effect of Acupuncture and EA for PHN

PHN belongs to a common kind of neuropathic pain and remains difficult to manage (20). Pharmaceutical therapies and surgical options are not applicable to all PHN patients and their efficacy is also often limited by unavoidable side effects (21). As a non-pharmaceutical therapy, acupuncture can not only relieve pain sensation of PHN but also improve anxiety and depression (22), which are common complications in PHN patients. Meanwhile, it also has the advantages of simple operation, less medical cost and negligible side effects.

Acupuncture has been widely used for treating PHN. Several previously published systematic reviews and meta-analysis (22, 23) indicate that acupuncture (including EA) is safe and may reduce pain intensity, relieve anxiety, and improve quality of life in PHN patients, but all currently available evidence remains inconclusive owing to the low methodological quality of included studies.

As one important modality of acupuncture therapy, EA is also frequently adopted for treating PHN. Previous clinical trials reveal that EA (24, 25) or in combination (26–28) with other treatments is beneficial for PHN. For example, a pilot RCT by Garridos et al. (27) found that the combination of EA and ketamine shows an early and long-term anti-hyperalgesic effect in PHN patients. A RCT conducted by Cao et al. (25) revealed that effect of EA alone for reducing pain intensity is more significant than Western medication (i.e., oxcarbazepine plus mecobalamine) in PHN patients. Nevertheless, limited to significant methodological flaws, the insufficient number of studies and potential publication bias, the reliability of previous results and current evidence is weakened, which is supported by a recently published systematic reviews and meta analysis (29). Further studies with more rigorous methodological quality and a larger sample size are urgently warranted. Thus, this multicentre, randomized controlled, and assessor-blinded trial is designed to further investigate the efficacy and safety of EA for PHN.

### Strengths of the Study

First, in this study EA is chosen as the acupuncture modality for investigation. Previous studies indicate that EA can increase pain threshold significantly and manage pain more effectively than traditional manual acupuncture (30, 31). The analgesia mechanism of EA is related to its activation of various bioactive chemicals including opioids, serotonin and norepinephrine, via peripheral, spinal, and supraspinal pathways (32). Despite that the exact mechanisms underlying EA for treating PHN are not very clear, several studies have investigated its possible mechanisms. One study (33) demonstrated that EA could inhibits neuron cell autophagy in PHN by increasing miR-223-3p expression, thereby achieve the analgesic effects. The study of Wu et al. (34) found that EA can alleviate the mechanical allodynia in PHN rats by reducing expression of vascular endothelial growth factor (VEGF) in the dorsal horn of the lumbar spinal cord. Wu et al. (35) revealed that EA can improve thermal perception by recovering TRPV1-positive sensory neurons and nerve terminals. Another study (36) indicated that EA can decrease primary afferent nerve sprouting in the spinal dorsal horn and neuropathic pain by changing a growth-permissive environment in spinal dorsal horn into an inhibitory environment through activation of μ-opioid receptors. Additionally, with regard to EA parameters, 2/100 Hz is adopted in our trial given that alternating-frequency EA wave is more well-tolerated and previous studies reveal that EA of alternating-frequency mode plays an important role in analgesia by evoking the release of dynorphin and endorphin (37). However, to date, there is a lack of high-quality RCTs investigating the efficacy and safety of EA for treating PHN.

Second, acupoint prescription is carefully designed in our trial. Primary acupuncture points include three parts, namely, local Ashi acupoints, Jiaji acupoints (EX-B2) and distal Xi-cleft points. Jiaji acupoints (EX-B2) are located 0.5 cun bilateral to the posterior midline in the dorsal thoracic and lumbar region, ranging from the first thoracic vertebra to the fifth lumbar vertebra. From the prospective of anatomical analysis, the anatomical position of Jiaji acupoints (EX-B2) corresponds to the posterior branch of the spinal nerve in the corresponding segment. Tissues of Jiaji acupoint area have widely distributed nerve endings, posterior branch of spinal nerve and sympathetic nerve trunk near vertebral and this can explain the neurophysiological basis of Jiaji acupoint (38). Mechanism researches have revealed that EA at Jiaji acupoints (EX-B2) can alleviate pain by restraining the incoming of pain signals directly in the spinal cord level, adjusting pain pathways, regulating neurophysiological functions, and enhancing local blood circulation (26). In addition, based on meridian system theory in traditional Chinese medicine, Xi-cleft points are often used for acute pain (39).

Therefore, the results of this trial will ascertain the credibility of current evidence regarding the efficacy and safety of acupuncture on PHN.

### Limitations

First, given to the characteristic of acupuncture procedure as well as the complete difference between the two therapies in two groups (i.e., pharmacotherapy vs. non-pharmaceutical therapy involving complex procedures), it is difficult to blind acupuncture operator and patients in our study. Nevertheless, the purpose of blinding aims to avoid the effect of subjective factors from the researchers and participants on the study results. With the attempt to minimize the subjective influence, in our study outcome assessment and statistical analysis will be implemented by a third party who is blinded to the grouping.

Second, sham acupuncture is not selected as a control in our study due to two major considerations: (1) owing that acupuncture is well-accepted and widely used in 3 sub-centers that enroll participants, the vast majority of participants have rich experience of acupuncture, even if a sham acupuncture group is designed, complete blinding of participant remains difficult to achieve in our study. (2) Previous studies reveal that all types of sham acupuncture currently available are not completely inert, regarding of penetrating or non-penetrating, thereby all current modalities of sham acupuncture have positive treatment effects (40–42). In this scenario, the treatment effect of verum acupuncture is likely to be underrated when compared with sham acupuncture (43). Therefore, sham acupuncture is not selected the control in our study. Nevertheless, when interpreting study results in the future, extra considerations should be given to the psychological (non-specific) effects of acupuncture that may influence the treatment effect, such as participants' expectations and the doctor–patient interaction in acupuncture procedures.

Third, due to the funding of this study, the follow-up period only covers 8 weeks. The longer-term therapeutic effect of EA on PHN needs further investigation. And only 3 centers involve patient enrollment.

## Conclusion

In conclusion, this protocol describes a multicentre randomized controlled, assessor blinded trial that aims to investigate the efficacy and safety of EA for treating PHN. The results will ascertain whether EA can relieve the pain and improve the quality of life and emotional disorders in PHN patients. The findings will be of great significance for clinical practice to identify a complementary modality for treating PHN.

## Ethics Statement

The studies involving human participants were reviewed and approved by the Ethics Committee of The Third Affiliated Hospital of Zhejiang Chinese Medical University. The patients/participants provided their written informed consent to participate in this study. Written informed consent was obtained from the individual(s) for the publication of any potentially identifiable images or data included in this article.

## Author Contributions

DH is responsible for this study. YS and HH designed the trial protocol and drafted the manuscript. XinwL and HT revised the manuscript. XingL and YL planned a data analysis solution. YC and LW participated in recruitment. All the authors have read, revised, and approved this version of the manuscript.

## Conflict of Interest

The authors declare that the research was conducted in the absence of any commercial or financial relationships that could be construed as a potential conflict of interest.
