# Supplementary material for: Efficacy of Electroacupuncture Therapy in Patients With Postherpetic Neuralgia: Study Protocol for a Multicentre, Randomized, Controlled, Assessor-Blinded Trial
Source: Front Med (Lausanne). 2021 May 21;8:624797. doi: 10.3389/fmed.2021.624797 (PMC8175774; doi:10.3389/fmed.2021.624797)
Supplement: Supplementary file 1 [file Data_Sheet_1.docx]

**Appendix 1. Multiple tests of Quantitative sensory testing (QST)**

| → Quantitative sensory testing (QST): |
| --- |
| ▪ thermic sensation: |
| ▪ cold and warm detection threshold (CDT + WDT) |
| ▪ thermic difference threshold (TSL) |
| ▪ cold and heat pain threshold (CPT + HPT) |
| ▪ tactile detection threshold |
| ▪ mechanical pain threshold |
| ▪ mechanical pain intensity |
| ▪ mechanical allodynia |
| ▪ wind up phenomenon |
| ▪ vibration threshold |
| ▪ pressure pain threshold |

**Appendix 2. The questionnaire of Zoster Brief Pain Inventory (ZBPI)**

Instructions:

People with shingles may have many kinds of pain or discomfort in the area of their shingles rash. These sensations may persist or come back in the area of the shingles rash even after the rash disappears When answering the following questions about pain, please include all kinds of pain in the area of your shingles rash, including pain triggered by air blowing on the skin, by clothing rubbing against the skin, or by hot or cold temperatures.

Do not include pain or discomfort that is unrelated to your shingles, such as low back pain, arthritis pain, or headache.

1. Have you had any pain caused by your shingles in the last 24 hours? (circle one number)

1. YES 2. NO

2. On the diagram, **shade** in the areas where you feel pain. **Put an “X” on the area that hurts the most.**


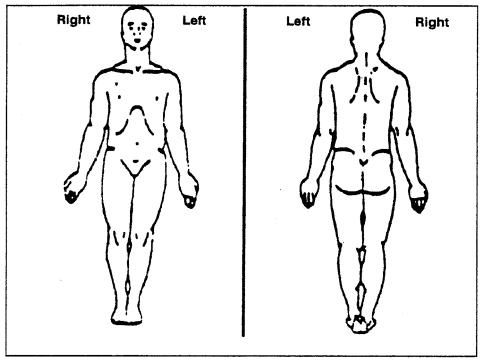


3. Please rate your pain by circling the one number that best describes your pain at its **worst** in the last 24 hours.

0 1 2 3 4 5 6 7 8 9 10

No pain Pain as bad

as you can imagine

4. Please rate your pain by circling the one number that best describes your pain at its **least** in the last 24 hours.

0 1 2 3 4 5 6 7 8 9 10

No pain Pain as bad

as you can imagine 5. Please rate your pain by circling the one number that best describes your pain on the **average** in the last 24 hours.

0 1 2 3 4 5 6 7 8 9 10

No pain Pain as bad

as you can imagine 6. Please rate your pain by circling the one number that tells how much pain you have **right now**.

0 1 2 3 4 5 6 7 8 9 10

No pain Pain as bad

as you can imagine 7. Are you receiving any treatments or medications for your **shingles pain**? (*Circle one number)*

1. YES 2. NO

8. In the last 24 hours, how much **relief** have these treatments or medications provided for your **shingles pain**? Please **circle** the one percentage that most shows

how much relief you have received.

0% 10% 20% 30% 40% 50% 60% 70% 80% 90% 100%

No relief Complete relief

9. **Circle** one number that describes how, in the last 24 hours, **shingles pain has interfered** with your:

A. **General activity**

0 1 2 3 4 5 6 7 8 9 10

Does not interfere Completely interferes

B. **Mood**

0 1 2 3 4 5 6 7 8 9 10

Does not interfere Completely interferes

C. **Walking ability**

0 1 2 3 4 5 6 7 8 9 10

Does not interfere Completely interferes

D. **Normal work** (including both work outside the home and housework)

0 1 2 3 4 5 6 7 8 9 10

Does not interfere Completely interferes

E. **Relations with other people**

0 1 2 3 4 5 6 7 8 9 10

Does not interfere Completely interferes

F. **Sleep**

0 1 2 3 4 5 6 7 8 9 10

Does not interfere Completely interferes

G. **Enjoyment of life**

0 1 2 3 4 5 6 7 8 9 10

Does not interfere Completely interferes
